# Improvement of Psychotic Symptoms and the Role of Tissue Plasminogen Activator

**DOI:** 10.3390/ijms161126053

**Published:** 2015-11-18

**Authors:** Silvia Hoirisch-Clapauch, Antonio E. Nardi

**Affiliations:** 1 Department of Hematology, Hospital Federal dos Servidores do Estado, Ministry of Health, Rio de Janeiro CEP 20221-903, Brazil; 2 Institute of Psychiatry, Federal University of Rio de Janeiro, and National Institute for Translational Medicine, INCT-TM CEP 22290-140, Brazil; antonioenardi@gmail.com

**Keywords:** cognition, refractory, resistant, schizophrenia, tissue plasminogen activator

## Abstract

Tissue plasminogen activator (tPA) mediates a number of processes that are pivotal for synaptogenesis and remodeling of synapses, including proteolysis of the brain extracellular matrix, degradation of adhesion molecules, activation of neurotrophins, and activation of the *N*-methyl-d-aspartate receptor. Abnormalities in these processes have been consistently described in psychotic disorders. In this paper, we review the physiological roles of tPA, focusing on conditions characterized by low tPA activity, which are prevalent in schizophrenia. We then describe how tPA activity is influenced by lifestyle interventions and nutritional supplements that may ameliorate psychotic symptoms. Next, we analyze the role of tPA in the mechanism of action of hormones and medications effective in mitigating psychotic symptoms, such as pregnenolone, estrogen, oxytocin, dopamine D3 receptor antagonists, retinoic acid, valproic acid, cannabidiol, sodium nitroprusside, *N*-acetyl cysteine, and warfarin. We also review evidence that tPA participates in the mechanism by which electroconvulsive therapy and cigarette smoking may reduce psychotic symptoms.

## 1. Introduction

Tissue plasminogen activator (tPA) is well known for its role in the coagulation pathway. Both endothelial and exogenous tPA convert plasminogen into plasmin. Plasmin dissolves the fibrin structure of thrombi, thus limiting thrombus formation to the site of vascular injury and restoring blood flow to ischemic territories [1].

Neurons, astrocytes, microglia, and oligodendrocytes also synthesize tPA. In these cells, tPA is stored in synaptic vesicles and released into the extracellular space by depolarization stimulus [2,3]. The expression of tPA is high in areas characterized by extensive remodeling of neuronal circuits throughout life, such as the hippocampus, the amygdala, prefrontal and cerebellar cortices, and the hypothalamus [3].

Until recently, it was assumed that once the brain was damaged, there was little, if any, possibility of axonal regeneration and formation of new synapses. Neurophysiological and neuroimaging studies support the notion that the human brain undergoes regeneration and synaptic plasticity. tPA plays an important role in both processes [4].

## 2. Tissue Plasminogen Activator and the Brain

Animal studies have demonstrated that tPA—itself or through activation of matrix metalloproteinases—mediates proteolysis of the extracellular matrix, which is a prerequisite for the formation and elimination of synapses, and for synaptic strength changes [5]. Both mechanisms underlie cognitive processes. Cognitive functions, which are related to the outcome of schizophrenia and are little influenced by antipsychotic treatment, depend on tPA-mediated synaptic remodeling [3,5,6]. Cognitive decline may precede the onset of psychosis in schizophrenia by almost a decade [7].

Apart from extracellular matrix proteolysis, tPA catalyzes a number of processes that are usually defective in psychotic patients. For example, by cleaving the NR1 subunit of the *N*-methyl-d-aspartate (NMDA) receptor, tPA increases calcium influx that enhances NMDA receptor signaling [3,8]. Calcium entry through the NMDA receptor determines whether neurons will die or survive: it seems that too much NMDA receptor activity is harmful to neurons, but so is too little [9]. NMDA receptor is a key element in excitatory transmission and synaptic plasticity. Evidence that aberrant NMDA receptor signaling contributes to schizophrenia pathogenesis comes from the fact that antagonists of NMDA receptor produce neurocognitive dysfunction, such as seen in schizophrenia [3].

Another mechanism dependent on tPA proteolytic activity is the cleavage of neurotrophins. Neurotrophins may have opposite functions depending on their state: pre-cleavage and post-cleavage. For example, brain-derived neurotrophic factor (BDNF) precursor binding to the p75 receptor causes a long-lasting reduction in synaptic strength—referred to as long-term depression, and to neuronal apoptosis. By contrast, binding of mature BDNF to its tyrosine kinase receptor leads to a long-lasting increase in synaptic efficacy—known as long-term potentiation, and to neuronal survival [10].

Dopaminergic transmission also seems to be influenced by tPA. Plasmin, acting on pre-synaptic dopaminergic neurons via plasminogen activator receptor (PAR)-1, enhances depolarization-evoked release of dopamine in the nucleus accumbens [11]. As such, tPA mediates emotional cognitive functions, especially reward-related memory reconsolidation [11].

## 3. tPA Inhibition

In the brain, tPA is inhibited by plasminogen activator inhibitor (PAI)-1 and by neuroserpin. PAI-1 is released by endothelial cells in the presence of inductors such as glucocorticoids, transforming growth factor-β, angiotensin, glucose, insulin, and triglycerides [12]. A single nucleotide polymorphism in the PAI-1 promoter—known as PAI-1 4G/5G, results in elevated PAI-1 levels and, consequently, in decreased tPA activity [13].

Little is known about neuroserpin gene activation, apart from it being post-transcriptionally regulated by triiodothyronine [14]. Point mutations in the neuroserpin gene may cause an uncommon form of dementia, named familial encephalopathy with neuroserpin inclusion bodies [15].

## 4. Conditions that Inhibit tPA Function Are Prevalent in Schizophrenia

Markers of low tPA activity consistently described in schizophrenia include hyperhomocysteinemia and antiphospholipid antibodies, such as lupus anticoagulant and IgM isotype anticardiolipin antibody [16,17,18]. Importantly, both hyperhomocysteinemia and antiphospholipid antibodies may affect tPA activity without affecting tPA levels [19].

Homocysteine, for example, inhibits tPA interaction with a heterotetramer formed by two annexin A2 molecules and two molecules of protein p11 (also known as S100A10). Since the heterotetramer increases tPA-mediated plasmin generation, hyperhomocysteinemia impairs tPA activity [19].

In the central nervous system, protein p11 interacts with the serotonin 5-HT1B receptor, which might explain the positive correlation between homocysteine levels and the severity of schizophrenia negative symptoms [18].

Our group has recently searched 70 patients with schizophrenia or schizoaffective disorder and 98 controls without mental disorders for markers of reduced activity of tPA. Hyperhomocysteinemia and antiphospholipid antibodies were highly prevalent in patients, but not in controls. Besides, we have identified a high prevalence of a not-previously described marker: free-protein S deficiency. Free-protein S and functional protein C are natural anticoagulants that form a complex, which inhibits tPA inhibitors. None of the controls had free-protein S deficiency and all participants had normal protein C levels, suggesting that protein S could have a role in schizophrenia independent of protein C [20]. In the same study, the association of the PAI-1 4G/5G polymorphism with hyperinsulinemia or hypertriglyceridemia was highly synergistic for acute episodes [20].

Based on the finding that chronic patients and those studied during acute episodes had more markers of low tPA activity (3–6 markers per patient, mean 3.1) than patients in remission (0–3, mean 0.9) or controls (0–2, mean 0.5), we have postulated that various concomitant conditions reducing tPA activity would be required for full expression of the disease [20].

It is possible that the number of markers per patient was underestimated. This is because some authors have noticed a negative correlation of anticardiolipin antibody levels with disease exacerbation, which is highly suggestive of antibody consumption during acute episodes [21].

Assuming that tPA has a crucial role in cognitive functions and that markers of low tPA activity are prevalent in patients with schizophrenia or schizoaffective disorder, it is not surprising that tPA plays an important role in the mechanism of action of pharmacological and non-pharmacological interventions that alleviate psychotic symptoms.

## 5. Lifestyle Interventions and Nutritional Supplements

### 5.1. Metabolic Interventions Effective in Decreasing Glucose and Insulin Levels

Since the PAI-1 promoter responds to glucose and insulin [12], one would expect that interventions effective in decreasing glucose levels and insulin synthesis, such as regular aerobic exercises and carbohydrate-restricted diets, could help normalize tPA activity. Confirming the expectations, obese patients with schizophrenia or schizoaffective disorder who achieve weight reduction with a program incorporating nutrition counseling and aerobic exercise may experience significant improvement of the mental symptoms [22]. Resolution of longstanding schizophrenia symptoms has been also reported after starting a low-carbohydrate, ketogenic diet [23].

Further evidence that interventions aimed at normalizing glucose and insulin levels may improve cognitive function comes from a study randomizing schizophrenia patients and healthy controls to exercise or non-exercise. Following aerobic exercise, hippocampal volume increased significantly in patients and healthy subjects, with no change in the non-exercising patients. Schizophrenia patients had modest cognitive improvement, which correlated with hippocampal volume changes [24].

### 5.2. Omega 3 Polyunsaturated Fatty Acids

Omega 3 polyunsaturated fatty acids increase tPA levels in the prefrontal cortex and hippocampus of rats submitted to chronic unpredicted mild stress to induce depressive behavior [25]. The impact of omega 3 in human tPA brain levels has not been determined, but omega 3 polyunsaturated fatty acids decrease serum triglycerides, with a magnitude proportional to triglyceride serum concentration [26]. Mice studies have shown that hypertriglyceridemia is an important element in the pathophysiology of learning and memory problems associated with obesity. The same studies have demonstrated that lowering triglyceride levels restores cognitive function [27]. Since the PAI-1 promoter responds to triglycerides, the reduction in PAI-1 levels observed in patients receiving omega-3 polyunsaturated fatty acids may be possibly related to the normalization of triglyceride levels [12,26].

One important trial supporting the hypothesis that omega-3 polyunsaturated fatty acids may influence cognition was conducted with young people at high risk of developing schizophrenia. This randomized, double-blind, placebo-controlled study has evaluated whether omega-3 polyunsaturated fatty acid supplementation for 12-weeks could prevent progression to psychotic disorder [28]. During a median of 6.7 years of follow-up, almost 10% of the omega-3 group developed psychosis compared to 40% of the patients who received placebo. Furthermore, the placebo group had an earlier onset of psychosis. Severe baseline negative symptoms increased the chances of treatment response [28].

### 5.3. Folic Acid, Vitamin B12, and Pyridoxine

Hyperhomocysteinemia may result from deficiency of folate, vitamin B12, pyridoxine, from heavy cigarette smoking, alcohol abuse or dependence, or renal dysfunction [29].

Methylenetetrahydrofolate reductase (MTHFR) recycles folic acid. The homozygous MTHFR C677T polymorphism, present in 10%–12% of the world population, results in greater than 70% reduction in enzyme activity [30,31]. Since folic acid is required to metabolize homocysteine, this homozygous genotype may increase plasma homocysteine levels in individuals with low folate intake. In a cohort of first-episode drug-naïve schizophrenia, folate levels were directly related to hippocampal volume, while for homocysteine levels the correlation was negative [18].

In genome-wide association studies taking into account the association of the MTHFR C677T polymorphism with plasma homocysteine levels, the effect of homocysteine plasma levels on schizophrenia diagnosis was over two-fold [30]. Reduced folate levels in the absence of increased homocysteine levels do not increase the risk of schizophrenia [31]. Prolonged folic acid, vitamin B12, and pyridoxine supplementation was effective in alleviating symptoms of schizophrenia individuals with extremely high homocysteine levels (around 24 μmol/L) [29].

## 6. Hormones

### 6.1. Pregnenolone

Progesterone binds to different receptors in the rodent brain. One of them is progesterone receptor membrane component 1 (PGRMC1, also known as 25-dx), which is found in microglia, astrocytes, and neurons [32]. Unlike other progesterone receptors, PGRMC1 is uniformly expressed in all hippocampal neurons [32]. In neurons and glia, progesterone is synthesized from pregnenolone [33].

In the ovary, progesterone has been shown to possess antiapoptotic properties. In granulosa cells, PGRMC1 interacts with PAI-1 RNA binding protein (PAIRBP1). Progesterone, competing with PAI-1 for the PGRMC1/PAIRBP1 complex, immortalizes the granulosa cells [34]. Although the relationship between PGRMC1 and PAIRBP1 in the brain has not been demonstrated yet, as it has been in the ovary, evidence that progesterone may afford protection against psychotic symptoms comes from relapses of schizophrenia symptoms, rare during pregnancy, but common in the postpartum period [35].

In randomized, double-blind, placebo-controlled trials, adjunctive pregnenolone reduced the severity of negative symptoms of schizophrenia and schizoaffective disorder, especially if the patient has not been treated with mood stabilizers [36,37]. More evidence that progesterone improves schizophrenia symptoms comes from atypical antipsychotics. Clozapine, and to a lesser degree olanzapine, elevates pregnenolone levels in the hippocampus and cerebral cortex of rats [38]. Additional studies are required to evaluate if the benefits of clozapine, olanzapine, and pregnenolone in the treatment of mental disorders depend on the competition between pregnenolone and PAI-1 for the PGRMC1/PAIRBP1 complex.

### 6.2. Estrogen

Estrogen receptors α and β have opposite effects on the human PAI-1 promoter: while β receptor suppresses it, α receptor induces it [39]. In the human hippocampus, estrogen receptor β levels are greater than estrogen receptor α levels [40].

The Definitive Oestrogen Patch Trial showed that estradiol may be an effective adjunctive therapy for women with treatment-resistant schizophrenia or schizoaffective disorder [41]. In this double-blind trial, females aged 18–45 years were randomized for transdermal estradiol 100 μg, 200 μg or placebo. Estradiol groups had greater symptom improvement than the placebo group, particularly in positive symptoms. The higher effect was seen with 200 μg. Benefits have also been seen in male schizophrenia patients [42].

### 6.3. Oxytocin

Oxytocin is another hormone that influences tPA activity in the brain. In multiparae, a rise in tPA antigen occurs shortly after oxytocin infusion is started [43]. Intranasal oxytocin may improve psychotic symptoms and social cognition in patients with schizophrenia [44].

## 7. Pharmacological Interventions

### 7.1. Dopamine D3 Receptor Antagonists

Dopamine D3 receptors (D3R), localized in extrastriatal regions, possess the highest affinity for dopamine of all known dopamine receptors [45]. Studies with mice lacking functional D3R or treated with novel, potent D3R antagonists have indicated that the receptor is a key player in neurogenesis and learning [46]. These mice had enhanced activation of cyclic AMP response element-binding protein (CREB), which is responsible for tPA and BDNF transcription. An increased immunoreactivity of tPA and mature BDNF in the prefrontal cortex and hippocampus of animals using D3R antagonists supports the hypothesis that tPA is involved in cognitive functions [46]. ABT-925, a selective D3 antagonist tested in phase II studies in schizophrenia patients, has been shown to enhance cognition [47].

### 7.2. Retinoic Acid

Many genes coding for proteins whose expression is altered in the prefrontal cortex of schizophrenia are either directly or indirectly regulated by retinoic acid, a metabolite of vitamin A. Examples include pyruvate kinase muscle isozyme, mitochondrial aconitase 2, hexokinase 1, malate dehydrogenase 1, gelsolin, neuron-specific enolase, and actinin α4 [48].

Long-term exposure of human astrocytes and endothelial cells to retinoic acid induces tPA expression in both cells and decreases PAI-1 expression in astrocytes [49,50]. While retinoic acid does not cross the blood–brain barrier [51], bexarotene, a substance that interacts with retinoid X receptors, does. A study controlling for antipsychotic agents showed that adjunctive bexarotene improved positive symptoms of schizophrenia and schizoaffective disorder, especially in patients with mean or high baseline positive scale scores and those who were not on lipid-reducing agents [52].

### 7.3. Valproic Acid

Valproic acid—a histone deacetylase inhibitor currently prescribed for the treatment of epilepsy, bipolar disease, and schizoaffective disorder—promotes neurite extension and neuronal growth in cultures of neurons and astrocytes [53]. The mechanism seems to involve a reduction in PAI-1 activity in astrocytes [53]. In cultured endothelial cells, valproate significantly increases tPA expression, while PAI-1 is only marginally affected by the treatment [54].

### 7.4. Cannabidiol

Δ9-tetrahydrocannabinol and cannabidiol are two psychoactive constituents of cannabis. Cannabidiol, an endocannabinoid modulator, decreases expression and secretion of PAI-1 [55]. While Δ9-tetrahydrocannabinol is psychotomimetic, cannabidiol reduces psychotic symptoms of schizophrenia [56].

### 7.5. Sodium Nitroprusside

After infusion, the vasodilator sodium nitroprusside is converted to the neurotransmitter nitric oxide [57], which mediates tPA release by endothelial cells [58]. In a double-blind, placebo-controlled trial, schizophrenia patients who received a single intravenous administration of low-dose sodium nitroprusside presented significant improvement in cognitive performance, whereas those who received placebo did not [59].

### 7.6. Warfarin

Warfarin has fibrinolytic properties that depend on its ability to increase tPA activity in the vascular compartment. Although it is not known whether warfarin increases tPA synthesis in cells other than the endothelium, it has been demonstrated that tPA crosses the intact blood-brain barrier [60]. Five patients with schizophrenia or schizoaffective disorder on chronic warfarin therapy for recurrent deep-vein thrombosis showed remission of psychotic symptoms [61]. Three of them had persistent antiphospholipid antibodies. The appropriate dose of warfarin may be difficult to establish, due to the interaction of the anticoagulant with foods, herbs, dietary supplements, and caffeine-containing beverages [62,63].

### 7.7. N-Acetyl Cysteine

In a double-blind placebo-controlled trial, adjunctive *N*-acetyl cysteine reduced negative symptoms in patients with chronic schizophrenia [64]. While some authors have reported that *N*-acetyl cysteine reduces lipoprotein (a) levels [65], others have found a significant decrease in homocysteine levels, but an insignificant decrease in lipoprotein (a) levels [66]. Since lipoprotein (a) increases PAI-1 expression in endothelial cells [67], a reduction in lipoprotein (a) levels would increase tPA activity by reducing PAI-1 levels or homocysteine levels.

## 8. Electroconvulsive Therapy

tPA is synthesized by chromaffin cells, stored in catecholamine–containing vesicles and co-released with catecholamines in response to sympathetic stimulation [68]. Electroconvulsive therapy (ECT) results in a 3- and 15-fold increase in norepinephrine and epinephrine levels [69]. Seizures induced by electroconvulsive shock are accompanied by increased plasma levels of tPA and up-regulation of tPA in the rat hippocampus [70].

Of note, the mechanism of action of ECT seems to involve a number of pathways facilitated by increased synthesis and release of tPA. These pathways include the activation of BDNF and vascular endothelial growth factor, increased NMDA receptor mediated signaling, increased zinc bioavailability, purinergic release, and pruning of dendritic spines [71].

ECT is frequently considered first-line treatment when a quick response is necessary, such as for patients with catatonia, aggression, and suicidal behavior [72]. Individuals with positive symptoms, such as paranoid delusions and hallucinations, also have a high probability of response [73]. The procedure is recommended for schizophrenia patients who did not respond to at least two trials of antipsychotic drugs of different classes, at adequate dosages for at least four weeks each, and for those who have shown intolerance to medication side effects [73].

## 9. Cigarette Smoking

In mice, single nicotine treatment significantly increases tPA activity in the extracellular space of the nucleus accumbens. Then, through PAR-1 activation, tPA and plasmin regulate dopamine release and nicotine-induced reward [74].

Cigarette smoking is highly prevalent in schizophrenia. Among first-episode patients, it has been reported that smokers have significantly lower severity of negative and depressive symptoms in comparison with non-smokers. Besides, psychosis onset occurs later in life in patients with severe nicotine dependence than in those with mild nicotine dependence or nonsmokers [75]. Smoking abstinence impairs attention and spatial working memory performance in smokers with schizophrenia, and smoking reinstatement reverses the impairment. Nicotine replacement therapy for schizophrenia patients who smoke very low nicotine content cigarettes may preserve cognitive function [76].

## 10. Future Directions

### 10.1. Intranasal tPA

In rats, tPA administrated intranasally during the subacute phase of traumatic brain injury or experimental stroke promotes neuroplasticity and significantly improves cognitive function [4]. Brain hemorrhage, a side effect of intravenous tPA, did not occur when animals were treated with intranasal tPA [77]. The role of intranasal tPA in psychotic disorders remains to be defined.

### 10.2. Rituximab

Patients with antiphospholipid antibody syndrome resistant to conventional medications may respond to treatment with rituximab, an anti-CD20 monoclonal antibody [78]. To date, no study has assessed the impact of rituximab in individuals with schizophrenia spectrum disorders refractory to antipsychotics.

## 11. Conclusions

tPA-mediated synaptic plasticity and neuronal regeneration are crucial processes for major brain functions. The hypothesis that tPA dysfunction might explain some obscure aspects of schizophrenia pathophysiology is supported by the antipsychotic armamentarium. The enhancement of tPA activity seems to be a common denominator of many interventions effective in mitigating psychotic symptoms and improving cognitive deficits, including lifestyle modifications, hormones, medications, electroconvulsive therapy, and even cigarette smoking. Controlled studies are needed to determine how interventions aiming specifically at correcting activity of tPA affect the outcome of psychotic disorders.
